# Diurnal variation of heart rate variability in individuals with spinal cord injury

**DOI:** 10.1186/s12938-024-01256-6

**Published:** 2024-06-20

**Authors:** Jittima Saengsuwan, Arphatsorn Ruangsuphaphichat, Lars Brockmann, Patpiya Sirasaporn, Nuttaset Manimmanakorn, Kenneth J. Hunt

**Affiliations:** 1 Department of Rehabilitation Medicine, Faculty of Medicine, Khon Kaen University, Khon Kaen, Thailand; 2 The Laboratory for Rehabilitation Engineering, Institute for Human Centred Engineering, Bern University of Applied Sciences, Biel, Switzerland

**Keywords:** Spinal cord injury, Heart rate variability, Diurnal variation, Cosinor model, Autonomic dysreflexia, SCI

## Abstract

**Background:**

Heart rate variability (HRV) may provide objective information about cardiogenic autonomic balance in individuals with spinal cord injury (SCI). The aim of this study was to characterize the diurnal variation of HRV in individuals with SCI at lesion level T6 and above and lesion level below T6.

**Methods:**

This was a retrospective analysis of a prior cross-sectional study. Individuals with chronic SCI underwent 24 h recording of the time between consecutive R waves (RR interval) to derive parameters of HRV as follows: standard deviation of all normal-to-normal R–R intervals (SDNN) and square root of the mean of the squared differences between successive R–R intervals (RMSSD) (time domain); and high frequency power (HF), low-frequency power (LF), very low frequency power (VLF), ultra-low frequency power (ULF) and total power (TP) (frequency domain). Changes in the magnitude of HRV outcomes over the 24 h period were investigated using a novel multi-component cosinor model constrained to the form of a three-harmonic Fourier series.

**Results:**

Participants were grouped as lesion level T6 and above (*n* = 22) or below T6 (*n* = 36). Most of them were male (*n* = 40, 69%) and the median age (interquartile range) was 50.5 (28) years. Both groups exhibited similar diurnal patterns in most HRV metrics. The lowest values occurred in the late afternoon (4–6 pm) and gradually increased, peaking around midnight to early morning (1–6 am). Exceptions included RMSSD, which peaked before midnight, and ULF, which showed a double peak pattern that peaked from 11 am to 1 pm and 4–6 am in participants with lesion level at T6 and above. The HRV values in participants with lesion level T6 and above were generally lower than participants with lesion level below T6, except for peak values of RMSSD, HF and LF.

**Conclusion:**

This study demonstrated substantial diurnal variation of HRV in participants with SCI in both groups of participants. In clinical and research settings, diurnal variations in HRV must be taken into consideration.

## Introduction

Autonomic dysfunction after spinal cord injury (SCI) is an under-researched area when compared to motor and sensory dysfunction [1]. Regarding cardiovascular autonomic dysfunction, cardiac sympathetic regulation is compromised in individuals with SCI in the cervical and upper thoracic regions. Individuals with lesion level T1 to T5 have impaired cardiac sympathetic regulation because the spinal sympathetic preganglionic neurons innervating the heart are disrupted; individuals with cervical level injury additionally have impaired bulbospinal input pathways, which provide an input to sympathetic preganglionic neurons [2, 3]. Individuals with lesions at or below the mid-thoracic level retain intact supraspinal sympathetic control of the heart and upper body vasculature [4]. On the other hand, cardiac parasympathetic regulation remains intact regardless of lesion level as this is regulated from vagal nuclei in the brain stem, which does not involve the spinal cord [5]. It was shown that the severity and neurological level of a lesion has a major impact on autonomic nervous system function [2]. For example, individuals with high-level SCI have lower maximal heart rate (HR) during exercise than their low-level SCI counterparts [6, 7]. The increase in heart rate is thought to be mainly due to parasympathetic withdrawal [1]. In addition, individuals with high-level SCI are prone to develop autonomic dysreflexia (AD), which is a common autonomic dysfunction characterized by sudden episodes of severe hypertension resulting from massive sympathetic discharge triggered by both noxious and non-noxious stimuli below the lesion level. This event is usually followed by baroreflex-mediated bradycardia due to increased cardiac vagal activity [8–10]. AD can result in a wide range of outcomes, from asymptomatic or mild discomfort cases to intracerebral haemorrhage or death [10]. Therefore, monitoring cardiac autonomic function after SCI is crucial and may be useful in clinical practice for predicting the development of autonomic dysfunction (including AD) and identifying patients at high risk for cardiac complications [11, 12].

Heart rate variability (HRV), which is the study of the variation in consecutive R–R intervals, is used as a non-invasive method to assess the relative shifts in autonomic cardiac control [13]. The information from HRV observations may be beneficial in the understanding of autonomic regulations in individuals with SCI. For example, the frequency domain method of analysis gives information based on power spectra delineated into four primary frequency bands: high frequency (HF, 0.15–0.40 Hz), low frequency (LF, 0.04–0.15 Hz), very low frequency (VLF, 0.0033–0.04 Hz) and ultra-low frequency (ULF, < 0.0033 Hz). HF power is thought to be mainly attributable to cardiac parasympathetic nervous system control and respiration [14]. LF power is mainly influenced by baroreceptor reflexes and both sympathetic and parasympathetic nervous system activity [14]. VLF power is thought to be mainly due to the heart’s intrinsic nervous system and thermoregulation, physical activity and renin-angiotensin and endothelial influences on the heart [15]. ULF power is less well studied, but is thought to be driven mainly by circadian rhythm [15]. Time-domain metrics include standard deviation of NN intervals, SDNN, and root-mean-square of successive RR interval differences, RMSSD. SDNN has been used to predict morbidity and mortality, and 24 h SDNN is highly correlated with ULF power and TP [15–18]. RMSSD reflects cardiac vagal activity and is correlated with HF power [14]; however, it is less affected by respiration than HF power [19].

HR, electrocardiogram waveforms, and HRV have marked fluctuations over the 24 h day [20, 21]. Twenty-four-hour rhythmicity in mammalian tissues and organs are driven by exogenous cycles of light and darkness, temperature, behavioural rhythms, such as the sleep–wake cycle, as well as endogenous circadian rhythmicity [22, 23]. Circadian rhythmicity of the cardiac cycle is controlled from endogenous origin by the suprachiasmatic nucleus of the hypothalamus [23]. Additionally, circadian rhythmicity is generated locally in the heart since it was found that cardiovascular tissues contain independent circadian clocks [24]. Different cardiovascular diseases have their onset at different times of day with most occurrences typically observed during the morning hours. These include myocardial infarction [25], supraventricular/ventricular arrhythmias [26–28], aortic dissection [29], stroke [30] and sudden cardiac death (SCD) [31, 32]. It is hypothesized that a shift towards increased sympathetic autonomic activation is responsible for this observation [20]. Diurnal variation in HRV may provide additional insights into the autonomic control of HRV in individuals with SCI.

There is limited research on how day vs. night variation affects the HRV of patients with SCI and it remains unclear to what degree the existence of diurnal variation in HRV is linked to patients’ autonomic function. Previous work has employed multiple-component cosinor analysis as a general modelling tool for diurnal data analysis [33]. In the present work, we refine this approach to a novel structure comprising a three-harmonic Fourier series that captures the 24 h duration of one day, the 12 h duration of the half-day, and also the 8 h and 16 h durations of the typical wake and sleep cycles. The aim of this study was to characterize the diurnal variation of HRV in individuals with SCI at lesion level T6 and above and lesion level below T6 using a three-harmonic Fourier series model.

## Results

### Participants

Seventy-two individuals participated in this study. During HRV data processing, there were 14 invalid data sets due to poor signal quality, leading to data sets of 58 participants being available for analysis. Among these, 22 individuals (37.9%) were classified as lesion level T6 and above and 36 individuals (62.1%) as lesion level below T6. Forty were male (69.0%). The median age was 50.5 years and the median duration after SCI was 5 years (Table 1).

**Table 1:** Demographic data of participants (*n* = 58)

Variables	At or above T6 (*n* = 22)	Below T6 (*n* = 36)	Total (*n* = 58)
Sex			
Male	15 (68.2)	25 (69.4)	40 (69.0)
Female	7 (31.8)	11 (30.6)	18 (31.0)
Age (years)			
Median (p25, p75)	48.5 (30, 63)	52.5 (33.5, 61)	50.5 (33, 61)
Range (years)	18–75	18–76	18–76
Completeness of lesion			
Complete (AIS A)	3 (13.6)	11 (30.6)	14 (24.1)
Incomplete (AIS BCD)	19 (86.4)	25 (69.4)	44 (75.9)
Cause of SCI			
Traumatic	12 (54.5)	14 (38.9)	26 (44.8)
Non-traumatic			
Degenerative	2 (9.1)	9 (25.0)	11 (19.0)
Inflammatory	5 (22.7)	4 (11.1)	9 (15.5)
Neoplastic	3 (13.6)	4 (11.1)	7 (12.1)
Infection	0 (0.0)	4 (11.1)	4 (6.9)
Other	0 (0.0)	1 (2.8)	1 (1.7)
Duration of SCI (years)			
Median (p25, p75)	5 (3, 9)	5.5 (2, 9.5)	5 (2, 9)
Range (years)	0.3 – 20	0.7 – 28	0.3 – 28
Underlying diseases			
No underlying disease	17 (77.3)	31 (86.1)	48 (82.8)
Hypertension	2 (9.1)	5 (13.9)	7 (12.1)
Dyslipidemia	4 (18.2)	5 (13.9)	9 (15.5)
Other	4 (18.2)	2 (5.6)	6 (10.3)
Medications			
Antihypertensive	5 (22.7)	7 (19.5)	12 (20.7)
Calcium channel blockers	2 (9.1)	5 (13.9)	7 (12.1)
Angiotensin converting enzyme inhibitors	1 (4.5)	1 (2.8)	2 (3.4)
Alpha-blockers	3 (13.6)	6 (16.7)	9 (15.5)
Angiotensin II receptor antagonists	0 (0.0)	1 (2.8)	1 (1.7)
Anticholinergics	14 (63.6)	18 (50.0)	32 (55.2)
Oxybutynin	13 (59.1)	14 (38.9)	27 (46.6)
Trospium	4 (18.2)	6 (16.7)	10 (17.2)
Medications for neuropathic pain	8 (36.4)	11 (30.6)	19 (32.8)
Gabapentin	5 (22.7)	11 (30.6)	16 (27.6)
Pregabalin	1 (4.5)	0 (0.0)	1 (1.7)
Tricyclic antidepressants	2 (9.1)	1 (2.8)	3 (5.2)
Carbamazepine	1 (4.5)	0 (0.0)	1 (1.7)
Antispastic	11 (50.0)	11 (30.6)	22 (37.9)
Baclofen	9 (40.9)	9 (25.0)	18 (31.0)
Tizanidine	2 (9.1)	1 (2.8)	3 (5.2)
Clonazepam	3 (13.6)	6 (16.7)	9 (15.5)
Diazepam	1 (4.5)	1 (2.8)	2 (3.4)

Data are presented as n (%) unless otherwise specified

Abbreviations: p25, 25th percentile; p75, 75th percentile

### Diurnal variations of heart rate and RR intervals

**Fig. 1:**
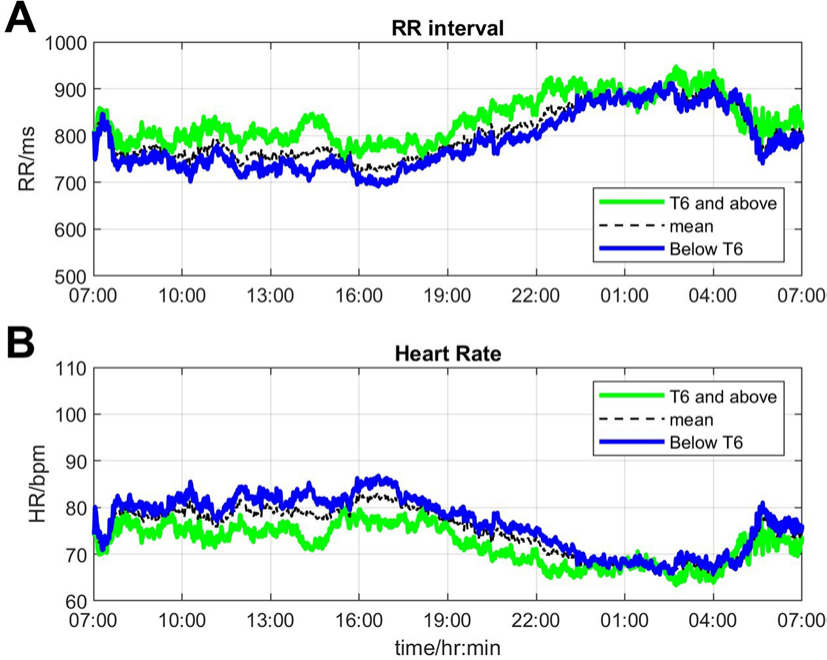
Twenty-four-hour variation of **A** RR intervals and **B** heart rate in individuals with lesion level T6 and above (green lines) and below T6 (blue lines). The dashed black lines are overall mean values for all participants irrespective of lesion level

Substantial diurnal variation was observed in RR intervals, heart rate, and all HRV metrics, in both groups of participants. RR intervals were generally shorter during the daytime compared to the night-time, which indicates slowing of heart rate during the night. The peak of RR intervals was at 2–4 am and they started to decrease thereafter. Overall, the RR interval was longer in participants with lesion level T6 and above implying a lower heart rate in this group of participants (Fig. 1).

### Diurnal variations of HRV

**Fig. 2:**
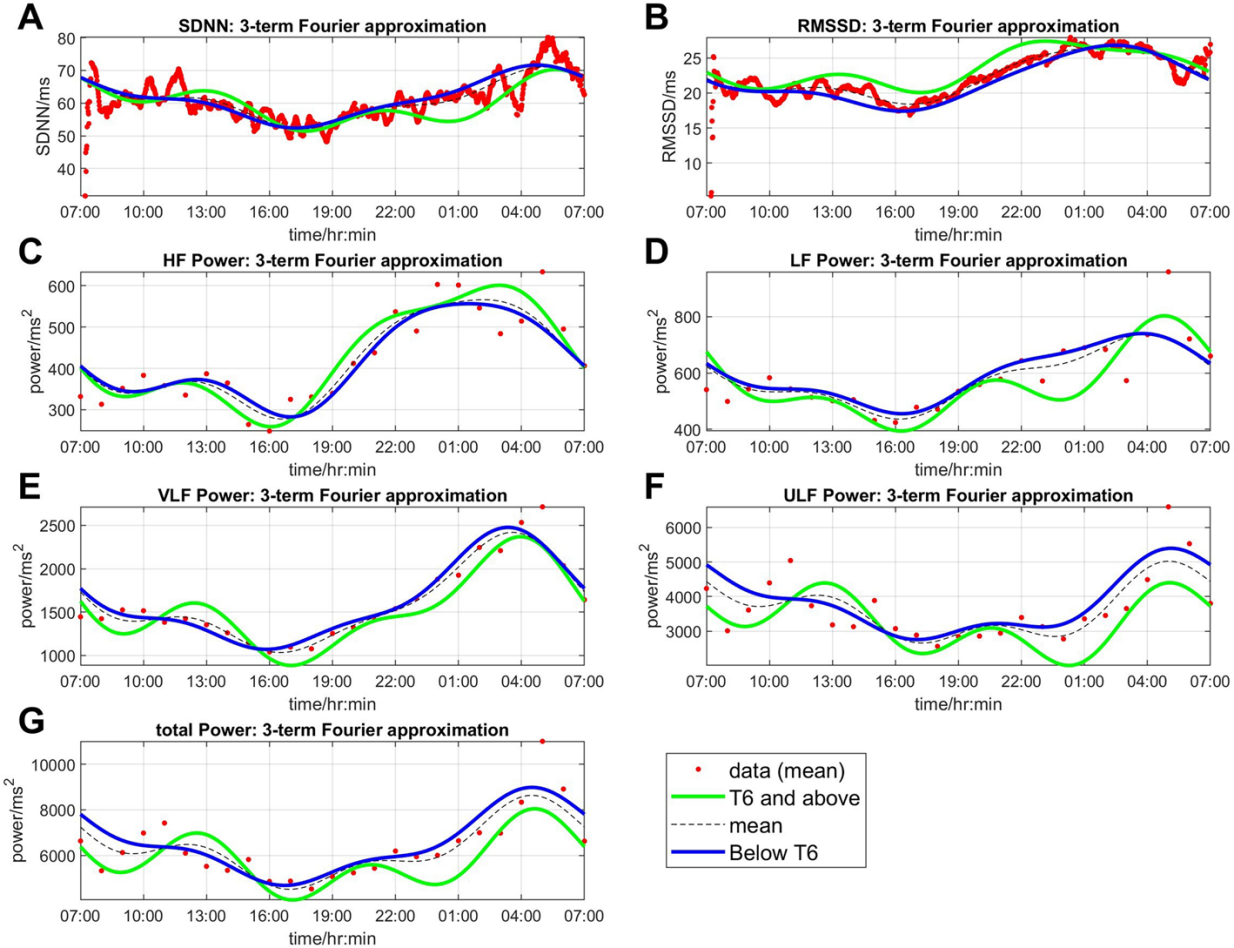
Twenty-four-hour variation of HRV: **A** SDNN, **B** RMSSD, **C** HF, **D** LF, **E** VLF, **F** ULF and **G** TP in individuals with lesion level T6 and above (green lines) and below T6 (blue lines). The lines represent the fitted mean values following Fourier regression. The red dots are overall mean data points and the dashed black lines are overall mean fits for all participants irrespective of lesion level

All HRV metrics were higher at night and lower during the day. In general, the lowest values occurred in the late afternoon (4–5 pm) and gradually increased thereafter to reach the highest peak around midnight to early morning. The highest peak typically occurred shortly before waking up time in the morning, except for RMSSD, which peaked before midnight and demonstrated a lower rate of decrease in the next 3–4 h compared to other HRV metrics (Fig. 2). The HRV values in participants with lesion level T6 and above were generally lower than in participants with lesion level below T6, except for peak values of RMSSD, HF and LF (Fig. 2).

Regarding the time domain metrics, SDNN and RMSSD showed the lowest values in the late afternoon (5–6 pm) and gradually increased to reach peak values thereafter. The peak of SDNN occurred in the morning (5–6 am) while the peak for RMSSD occurred much earlier, shortly before midnight (11–12 pm) in participants with lesion level T6 and above.

For frequency domain metrics, HF, LF and VLF reached their lowest values at approximately 4–5 pm. HF reached its peak value at 1–3 am while LF and VLF reached their peak values at 3–5 am. Participants with lesion level T6 and above showed higher variation in the magnitude of change in HF and LF (higher amplitude) compared to participants with lesion level below T6. ULF showed a different pattern: participants with lesion level T6 and above reached its trough at midnight–1 am while participants with lesion level below T6 reached the lowest level at around 4–6 pm. Additionally, participants with lesion level T6 and above showed a double peak pattern in ULF, whereby the peak values occurred at 11 am–1 pm and 4–6 am, while participants with lesion level below T6 showed only one peak value at 4–6 am. Since ULF had the highest power in the frequency domain metrics, TP characteristics corresponded to ULF, but consistently showed just one peak at 4–6 am in both lesion levels.

## Discussion

This study aimed to characterize the diurnal variation of HRV in individuals with SCI at higher (T6 and above) and lower (below T6) lesion levels. HRV exhibited substantial diurnal variation irrespective of lesion level. The timing of the lowest points and peaks of the HRV metrics were remarkably similar between groups, indicating that the rhythmicity of HRV shares a degree of similarity.

All HRV metrics in the present study exhibited diurnal rhythmicity, with a night-time peak and daytime nadir. The pattern was similar to that observed in able-bodied participants [34, 35]. Interestingly, previous studies observed that the peaks of SDNN, RMSSD, HF, LF and VLF occurred slightly later than ours (5–7 am) [34–36]. This difference may be explained by the early sleep and wake-up time of our participants in the inpatient unit, as sleep was found to significantly influence diurnal variation [37]. It is important to note that the diurnal variation observed here, while resembling that of able-bodied participants, does not necessarily imply normal autonomic function, as our HRV values were much lower than those found in able-bodied participants [38]. The diurnal variation in HRV established in this study highlights the importance of considering time of day in HRV research. Morning assessments generally show higher HRV values compared to afternoon assessments, and this should be accounted for study design and data interpretation.

Previous studies have reported lower or absent LF in individuals with cervical SCI compared to those with low paraplegia (below T6) [2, 39–42]. This was formerly interpreted as indicating low sympathetic activity. However, it has been demonstrated that LF is primarily influenced by baroreflex function [43], and may be influenced by both the parasympathetic and sympathetic divisions of the autonomic nervous system [14]. We could identify LF in all individuals in this study. The observed diurnal variation in LF values, which peaked at night and reached a nadir during the daytime in participants with SCI, was consistent with findings in able-bodied adults. However, the LF values were generally lower in our participants, and the peak occurred slightly earlier than reported in previous studies [34, 36]. Prior research has shown that lower HRV values and blunted diurnal variation are found in patients with conditions such as uncomplicated coronary artery disease and stroke [36, 44]. Interestingly, we found that participants with lesion level at T6 and above, who are known to have a higher risk of autonomic dysfunction, had lower daytime LF values but higher night-time LF peak values compared to participants with lesion levels below T6. This finding may reflect greater fluctuation in cardiac autonomic function in this group of patients.

The difference in daytime and night-time values was also found in HF and RMSSD. HF and RMSSD were reported to have lower values in participants with tetraplegia or high paraplegia (T1–T6) compared to low paraplegia [45] in most studies with short-term HRV. However, studies that measured 24 h HRV showed conflicting results, with either no significant difference [46] or higher HF and RMSSD [2, 47] in participants with tetraplegia or high paraplegia. Our study showed that participants with lesion level T6 and above had higher values of HF and RMSSD, especially in the afternoon and at night [39]. Since RMSSD and HF mainly reflect parasympathetic activity [14], the higher values found in participants with lesion level T6 and above may correspond to the lower baseline heart rate observed in this group of patients in our study. The difference in the day and night-time values may account for the discrepancy with the findings in previous studies.

Studies regarding the diurnal variation of VLF and ULF are limited. Korpelainen et al. demonstrated that VLF showed a daytime nadir and high values at night, reaching its peak value at 7–9 am in the morning in able-bodied adults [36]. Our study demonstrated a consistent pattern; however, our peak value occurred much earlier (3–5 am) than in the previous study. ULF was believed to be predominantly driven by the circadian rhythm, although other factors such as core body temperature, the renin-angiotensin system, and metabolism may also influence ULF [14]. There is no previous literature regarding the diurnal variation of ULF. However, ULF appears to exhibit the greatest difference between participants with lesion level T6 and above and those with lesion below T6. Further exploration of ULF is warranted.

We observed diurnal changes in RR intervals and, correspondingly, heart rate. Overall, participants with lesion level T6 and above exhibited longer RR intervals (indicating a lower heart rate) than those with lesion level below T6. Furlan et al. reported longer RR intervals in participants with motor complete lesions T6 and above compared to lesions below T6 in the acute phase (< 72 h) after SCI [48]. Moreover, multiple studies reported higher prevalence of bradycardia in the acute and subacute phase of SCI (within 4–6 weeks after SCI), especially in individuals with tetraplegia; however, this problem resolved in the chronic phase of SCI [49, 50]. Although the prevalence of bradycardia was comparable in chronic SCI, Claydon et al. demonstrated that in participants with chronic SCI, those with tetraplegia showed longer RR intervals in both supine and upright postures compared to participants with paraplegia (T2-T11) [40]. The lower heart rate observed in participants with lesion level T6 and above found in our study is consistent with a previous study which showed that the 24 h heart rate in individuals with low paraplegia (lesion level T6-T12) was significantly higher than in individuals with tetraplegia [47]. Furthermore, Revensbergen et al. demonstrated that the mean resting heart rate was 59 bpm in individuals with tetraplegia, 75 bpm in those with high thoracic lesions (T1–T5) and 77 bpm in those with low level lesions (T6 and below) 5 years after discharge [1]. The difference found in heart rate may be explained by an autonomic imbalance of heart regulation after SCI. In cases of high-level SCI where the spinal sympathetic pathways responsible for controlling heart rate are disrupted, parasympathetic vagal tone may predominate and lead to a lower heart rate [8, 49, 50].

This study had several limitations. The exclusion of participants with heart, neurological, or endocrinological diseases such as previous myocardial infarction or diabetes mellitus may lead to overrepresentation of healthier individuals. The evaluation of the respiratory rate, which needed to be less than 20 breaths/min as per the inclusion criteria, was conducted while the patient rested on the bed during the daytime. Therefore, we could not confirm that it would remain below this threshold throughout the entire 24 h period. Additionally, we did not have data regarding sleep-disordered breathing, which may impact nighttime HRV [51]. It was a single-centre study, and the lack of HRV data in participants with complete tetraplegia limits the generalizability of the results. Since age and sex affect diurnal variation of HRV [35], future studies with larger populations, classified by age, sex, lesion level and completeness of lesion, may provide more information. Furthermore, future studies focusing on the relationship of HRV and autonomic dysreflexia (AD), as well as HRV changes during AD, may provide additional insight into changes in cardiac autonomic regulation and offer further methods for early detection and management of the condition.

## Conclusion

This study demonstrated substantial diurnal variation of HRV in participants with SCI. Despite differences in lesion level, the diurnal patterns of HRV were remarkably similar between these groups and to those observed in general populations. In clinical and research settings, diurnal variations in HRV must be taken into consideration.

## Methods

### Participants

This is a retrospective analysis of HRV data collected from 72 participants derived from a prior cross-sectional study to determine test–retest reliability of long term HRV in spinal cord injury [52]. The study was conducted from October 2019 to August 2020. Ethical approval was obtained from the Khon Kaen University Ethics Committee in Human Research (HE621279). The studies were conducted in accordance with the Declaration of Helsinki. The participants provided their written informed consent to participate in this study.

Participants were individuals with SCI who were admitted for their annual urological surveillance at the University Hospital in Khon Kaen which is in the Northeastern region of Thailand. Inclusion criteria were SCI more than 3 months and age ≥ 18 years. Exclusion criteria were abnormal breathing pattern (respiratory rate > 20 breaths/min or < 10 breaths/min), fever (body temperature ≥ 37.8 degrees Celsius), concomitant cardiac or neurological diseases as well as endocrine disorders including diabetes mellitus and thyroid diseases.

### Study protocol

Clinical data such as diagnosis, duration after SCI, underlying diseases and current medications were retrieved from the participants’ medical records. The neurological level of injury and degree of impairment after SCI were assessed based on the International Neurological Classification of Spinal Injury using the American Spinal Injury Association Impairment Scale (AIS) [53]. HRV recording was performed for 24 h starting at approximately 8 am. Individuals refrained from smoking, drinking caffeine or alcohol for 24 h before measurements. The recordings were conducted prior to the urological check-up. Patients were instructed to perform their normal daily activities as usual. Since the patients in this study were admitted in the inpatient rehabilitation, their wake-up time, sleep time and mealtimes were comparable. The RR interval recordings were obtained using a wearable heart rate sensor and wristwatch monitor (Polar H10 and V800, Polar Electro Oy, Kempele, Finland) which uses a sampling rate of 1 kHz [54]. The sensor was attached using a chest belt. Since the prior study was to establish the test–retest reliability of the long-term HRV recordings, two data sets were available for each participant. For the HRV analysis, the data sets were randomized to select one 24 h period for each participant.

### Outcomes and data processing

The data were exported as a text file to HRV analysis software that was implemented in Matlab (The Mathworks, Inc., USA). Data which had signal recording of less than 24 h or showed a noisy signal, signal gap or multiple skipped measurements were excluded from the analysis; full details can be found in the Supplementary Materials of reference [52]. The remaining data sets were preprocessed for artefact detection and removal. Artefact detection was performed using two methods previously described [52]: (i) maximal and minimal values for plausible RR values were defined (min = 400 ms, max = 1650 ms), (ii) the difference between two successive RR intervals was set to be at a maximum of ± 20% of the previous value.

The outcome parameters consist of both time domain (SDNN and RMSSD) and frequency domain (HF, LF, VLF, ULF, TP) metrics which were processed to give 1 min and 60 min averages, respectively. The Lomb–Scargle least squares spectral analysis method for spectral density estimation was used, as it is optimised for non-uniformly spaced data sets such as RR time series.

We used a multi-component cosinor model [33] that we constrained to the form of a three-harmonic Fourier series. This model has a constant component (known traditionally as the direct current, or DC term) and three cosine terms of increasing frequency, i.e. the first harmonic (fundamental mode), and the second and third harmonics, viz.

1 $$f\left(t\right)={A}_{0}+{\sum }_{n=1}^{3} {A}_{n}\text{cos}\left(n\omega t+{\varphi }_{n}\right),$$

where ${A}_{0}$ is the DC term, ${A}_{n}$ and ${\varphi }_{n}$ are the harmonic amplitudes and phase angles, respectively, and $\omega =\frac{2\pi }{T}$ is the fundamental frequency, with $T$ being the period. The rationale for this novel model structure is that the fundamental frequency captures the 24 h duration of a day, the second harmonic represents the 12 h period of a half-day, while the third harmonic period of 8 h captures the fact that the sleep and wake cycles have durations of approximately 8 and 16 h, respectively.

The Fourier series can also be expressed in sine–cosine form as

2 $$f\left(t\right)=\frac{{a}_{0}}{2}+{\sum }_{n=1}^{3}\left({a}_{n}\text{cos}\,n\omega t+{b}_{n}\text{sin}\;n\omega t\right).$$

Transformation from the form of Eq. (2) to that of Eq. (1) is obtained using.

3 $${A}_{0}=\frac{{a}_{0}}{2}, {A}_{n}= \sqrt{{a}_{n}^{2}+{b}_{n}^{2}} , \; {\text{and}} \; {\varphi }_{n}= {\text{tan}}^{-1}\left(\frac{{-b}_{n}}{{a}_{n}}\right).$$

### Statistical analysis

Categorical variables are presented as numbers and percentages. Normality of data was tested with a Shapiro–Wilk test. Continuous parameters are presented as median and interquartile range (IQR) because the data were not normally distributed. The analyses were perform using SPSS (IBM SPSS Statistics for Windows, Version 28.0. Armonk, NY: IBM Corp).

## Data Availability

The datasets used and analysed during the current study are available from the corresponding author on reasonable request.
